# Coral community data Heron Island Great Barrier Reef 1962–2016

**DOI:** 10.1038/s41597-022-01747-y

**Published:** 2022-10-12

**Authors:** Jason E. Tanner, Joseph H. Connell

**Affiliations:** 1 South Australian Research and Development Institute, PO Box 120, Henley Beach, SA 5022 Australia; 2 Department of Ecology, Evolution and Marine Biology, University of California, Santa Barbara, California 93106 USA

**Keywords:** Community ecology, Community ecology

## Abstract

Here we describe benthic composition data derived from benthic photoquadrats collected over 41 surveys between 1962 and 2016 at four sites on Heron reef, at the southern end of Australia’s Great Barrier Reef, to assess change in coral composition over time. Surveys have often been annual, in a few years sub-annual, and the longest gap is six years. A subset of the data from two sites with the most complete records has been fully processed to allow the size of all individual colonies, and changes in species composition and cover, to be tracked over time. The taxonomy in these quadrats has been carefully checked for internal consistency, and is generally at the species level. A second subset has been processed, but has not been through full quality control, while a third subset exists as images only. This is the longest, 56 years, regular photographic record of coral cover in existence, and provides a valuable temporal contrast dating back in time to more recent studies of greater geographic extent and/or resolution.

## Abstract (summary)

**Table:** 

Measurement(s)	coral community composition • Coral colony area • Coral colony perimeter
Technology Type(s)	still camera (film and digital)
Sample Characteristic - Organism	Anthozoa
Sample Characteristic - Environment	Coral reef
Sample Characteristic - Location	Australia • Heron Island

## Background & Summary

**Fig. 1:**
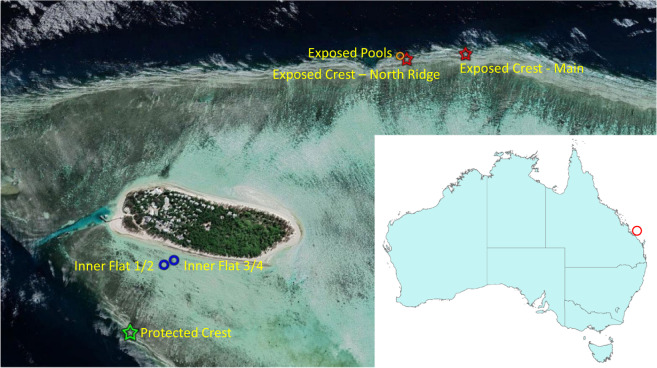
Map showing the location of Heron Island (red circle on inset), and the study sites described here. Colour coding indicates subsites grouped together as a single site, stars indicate sites still extant in 2019 and circles indicate site where the marker stakes had been lost prior to 2019. Main image source: Google Earth.

We describe here a unique long-term data set describing coral cover in a series of permanent photo quadrats established on Heron Island Reef since 1962. Heron Island (23^o^26′ S, 151^o^55′ E) is located at the southern end of Australia’s Great Barrier Reef, around 65 km offshore from the mainland, and is surrounded by a broad platform reef. A series of quadrats were established by JH Connell at four intertidal or shallow subtidal locations across the reef (Fig. 1), representing different environments and levels of exposure to cyclones. These quadrats were monitored close to annually up until 2018, and in some early years sub-annually, although some sites were lost earlier due to loss of marker stakes. Photographs from one survey in each year up until 2012 have been orthorectified, and manually digitised to map the outline of each coral colony present, thus allowing the area of each colony to be calculated. The data set thus includes both percent cover, by coral species, as well as the size distribution of colonies. Full data for the exposed and protected crests are presented here, these being the two sites still extant. The exposed pools and protected inner flat sites have been lost, and the data sets for these have not been through full QA/QC, so only the images and shapefiles are included.

At each site, different numbers of permanent quadrats were established. At most sites, the quadrats were contiguous, so do not constitute independent replicates. However, this should be viewed in the context of the unique temporal replication and taxonomic resolution^1^, and the difficulties in collecting data such as this prior to the advent of digital cameras.

Observations and data derived from earlier parts of this study have been influential in the development of ecological theory. In particular, they featured in the formalisation of the intermediate disturbance hypothesis^2^, as well as the Connell-Slatyer model of ecological succession^3^. More detailed analysis of earlier parts of these, and related data sets, explore the role of disturbance^4,5^ and competition^6^ in coral dynamics. Data from the study have also been used in a number of modelling studies of coral community dynamics^7–11^. These previous analyses were based on an independent extraction of data from the images using hand drawn paper maps. An analysis of the exposed and protected crest data sets presented here suggests that full recovery in both species composition and size distribution can occur over decadal time scales after a major cyclonic disturbance despite the removal of all corals and the alteration of the drainage pattern of the reef flat. However, if environmental factors don’t return to pre-disturbance conditions, then recovery will not necessarily occur^1^.

Given that this is the only study of coral communities with such temporal length and resolution, as well species level taxonomic resolution, the data are likely to be valuable in helping to place more recent studies with higher geographic resolution and/or extent into a better historical context. Full documentation of the data set will also allow others to continue the study on into the future, further building on Prof. Connell’s legacy.

## Methods

### Study site and field data collection

**Fig. 2:**
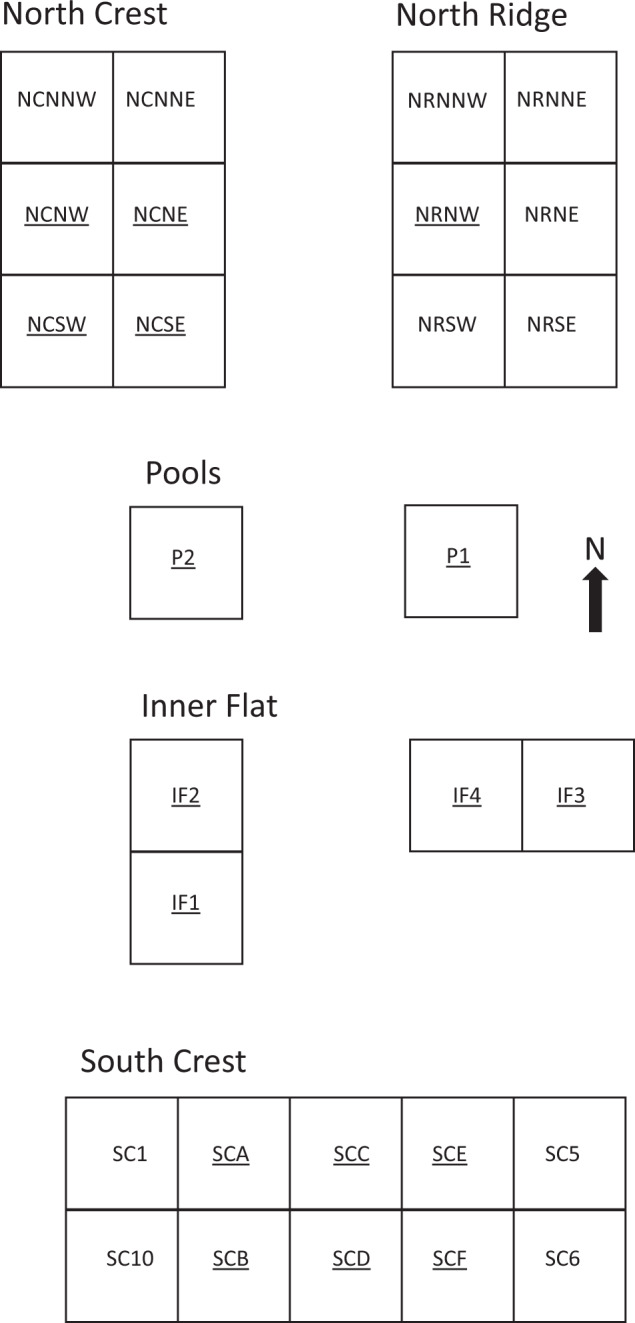
Quadrat layouts for each of the four sites respectively, noting that the north crest and north ridge have been treated as a single north crest site in previous publications. Underlining indicates original 1962/63 quadrats. Other quadrats were added in or after 2008, as indicated in the text. Contiguous quadrats are pictured bordering each other. Spacing between separate quadrats or groups of quadrats is not shown to scale. Note that up until 2005, NRNW was known as NR. The acronyms in each quadrat represent its name.

Permanent 1 m^2^ photoquadrats were established on Heron Reef in 1962/63, using 9 mm diameter mild steel (rebar) pegs, which were replaced over time. From the 1990’s, replacement pegs were stainless steel for greater longevity. Four sites were established, the protected (south) crest, inner flat, exposed (north) crest and exposed pools. Co-ordinates for each site are presented in Table 1, the layout shown in Fig. 2, and sites have been well described previously^5,6^. At each census, a 1 m^2^ frame divided into a 5 × 5 grid using string was placed over the pegs, and the quadrat photographed from directly above at low tide. From 1963 until 2003, a 35 mm camera and colour slide film were used. The camera was attached to a tripod affixed to the 1 m^2^ frame, and captured around 2/3 of the quadrat. The frame (and camera) were then rotated 180 degrees to capture the remainder of the quadrat. After 2003, a hand-held digital camera was used, with the entire quadrat being captured in a single image. Concurrent with each census, mud maps of each quadrat were hand drawn in the field, and all colonies identified *in situ* by someone with expertise in coral taxonomy.

**Table 1:** Coordinates of the study sites on Heron Island Reef (WGS84).

Site	Sub-site	Latitude	Longitude	Status as at 2019
Protected crest		−23.44693333	151.9124167	Extant
Inner flats	1/2	−23.44426667	151.91345	Lost in 2014
Inner flats	3/4	−23.44408333	151.91385	Lost in 2014
Exposed crest	Main	−23.43425	151.9273167	Extant
Exposed crest	North ridge	−23.43453333	151.92435	Extant
Exposed pools		−23.434297	151.924044	Lost in 2008

At the protected (south) crest, a set of six contiguous quadrats were established in 1963 in a 2 × 3 arrangement parallel to the waterline, and about 420 m southeast of the island. This site is exposed at low tide, and was photographed once all water had drained off it. Images of quadrats A, C & E (the shoreward row) from 1963 to 2012 have been fully processed, and the data have been through QA/QC. Data after 2012 exist as images only. These quadrats form the basis of previous analyses^1,4–6^ for this site. Photographs are available for quadrats B, D & F, but apart from 2003–2010, have not been processed. In 2010, an additional two quadrats were established either side of the original six, leading to a 2 × 5 arrangement. Again, only imagery is available for these additional quadrats.

At the inner flat, two pairs of contiguous quadrats were established in 1962, 44 m apart, about 70 m south of the island. This site is covered by ~10 cm of water at low tide, so could only be photographed on a still day. Imagery for this site is only available to 2012, after which the marker stakes appear to have been removed in a cleanup of the area. Images for one quadrat in each pair have been processed, but have not been subject to full QA/QC.

At the exposed (north) crest main site, a set of four contiguous quadrats was established about 1100 m northeast of the island in 1963. An additional single quadrat (north ridge) was established 326 m to the east. Images from 1963 to 2012 have been fully processed, and the data have been through QA/QC. Data after 2012 exist as images only. In 2005, the single north ridge quadrat was expanded to 4 m^2^, and in 2008, both subsites were expanded to six quadrats in a 2 × 3 arrangement. These additional quadrats have been digitised up to 2012, but have not been through full QA/QC.

The exposed pools are two individual quadrats about 5 m apart about 30 m north of the eastern (north ridge) exposed crest site. These are on the edge of a natural pool, and range from ~5–50 cm deep at low tide, and so could only be photographed on a calm day. Imagery for this site is only available until 2005, after which the marker stakes could not be relocated. Images from 1963 to 1998 have been processed, but have not been through full QA/QC.

### Retrieval of coral composition data from the photoquadrats

**Fig. 3:**
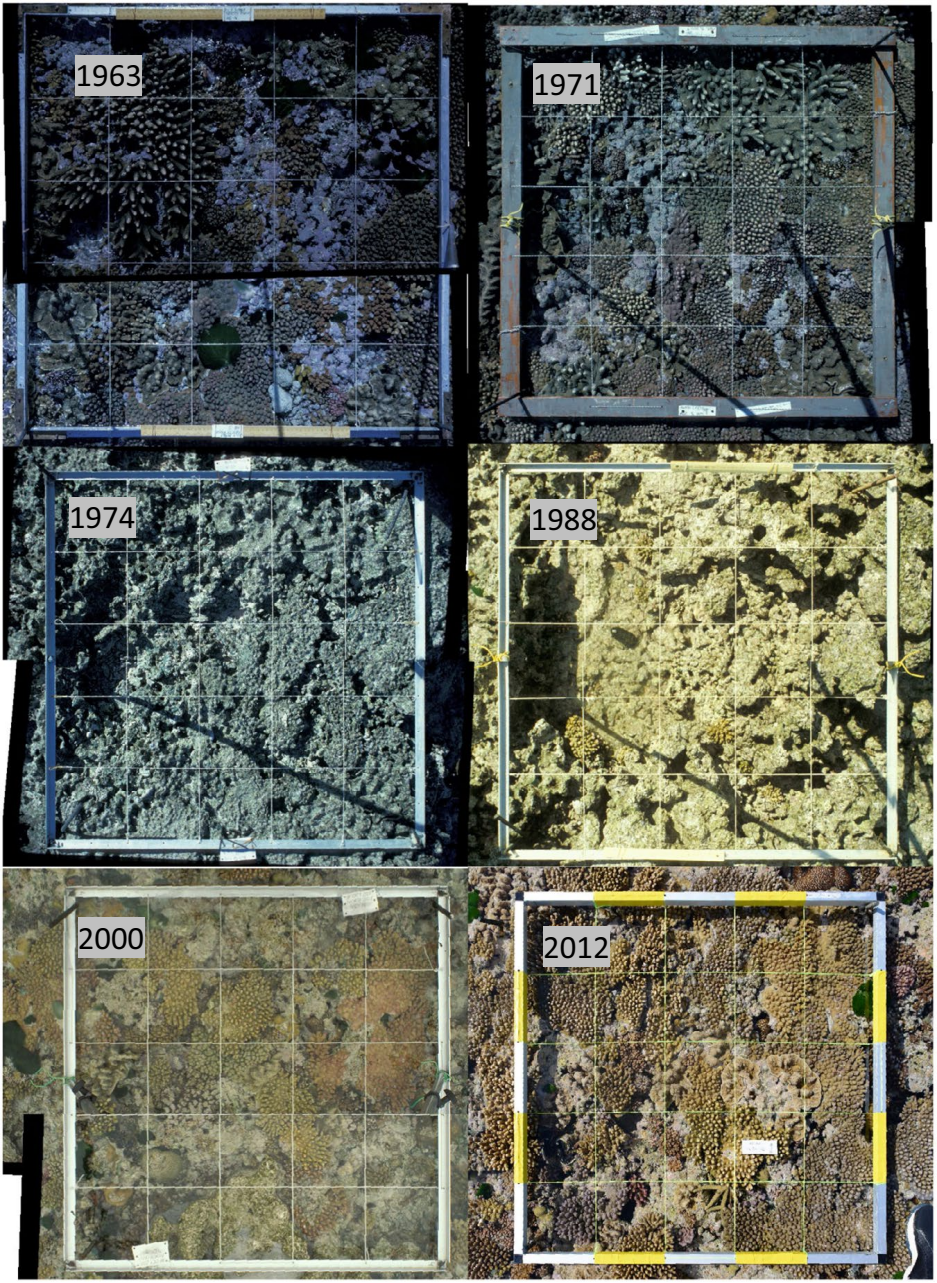
Example orthorectified and stitched (prior to 2001) images from the NCNE quadrat, showing the effects of a cyclone that removed all colonies in 1972, and slow recovery over subsequent decades.

Processing of the images involved scanning the colour slides to produce digital images, and then orthorectifying each image to a 1 m^2^ basemap in ArcGIS (ESRI Ltd). The corners of the frame, and the holes for the string grid, were used as control points for the orthorectification. For images that originated as colour slides, each half of the quadrat was individually orthorectified to the same basemap, producing a single image of the entire quadrat (see Fig. 3). While contiguous quadrats were orthorectified individually, they were done so against a basemap containing all quadrats in the group, meaning that the resulting images can be easily merged to create a single image of the group. The outlines of all visible coral colonies (>~1 cm^2^), and other benthic organisms such as algae and clams, were then digitised in ArcGIS to create a single shapefile for each quadrat for each year. Each colony was represented as an individual feature within the shapefile, and was assigned a unique colony number and species based on the mud maps drawn in the field. Colony numbers were consistent across years, allowing individual colonies to be tracked over time. If a colony underwent fission, the original colony number was retained for each, with the addition of a unique identifier after a decimal point. For example, if colony 35 split in two, the resultant colonies were identified as 35.1 and 35.2. If 35.2 later split again, the resultant colonies were identified as 35.2.1 and 35.2.2. If the colony overlapped the edge of the quadrat, only the area within the quadrat was digitised, and a flag was applied to indicate that only part of the colony was included (edgestatus = 1 in the data). Upon completion of digitisation, ArcGIS was used to calculate the area and perimeter of all colonies. While multiple census were conducted in 1963, 1971 and 1983, only a single census in each year has been processed. There are currently no plans to undertake further digitisation or QA/QC of this data set.

## Data Records

The data are available on figshare^12^ and are detailed in Tables 2–5. All scanned and digital slides are provided as jpeg files (.jpg) along with accompanying files created during the orthorectification process (.jgwx,.jpg.aux.xml, and.ovr). The results of the digitisation process are provided as shapefiles (.shp) and their accompanying files (.dbf, .shn, .sbx and .shx). File names include the quadrat name (as per Fig. 2), the year, and sometimes the month and day the image was taken. For scanned slides, there are generally two images for each date, labelled N and S, or E and W, indicating the cardinal direction of each half of the quadrat. For any images not orthorectified, shapefiles for the basemaps are also provided. The final processed data set is provided as a single csv file for each quadrat, with the fields detailed in Table 6. The shapefiles also contain colony perimeter, and several other parameters that were used for internal processing that can be ignored.

**Table 2:** Data availability for the protected (south) crest^12^.

	SCA	SCC	SCE	SCB	SCD	SCF	SC1	SC5	SC6	SC10
1963 Jan 3	s	s	s	s	s	s				
1963 Mar 24	s	s	s	s	s	s				
1963 Jul 3	s	s	s	s	s	s				
1963 Oct 2	s,f,q	s,f,q	s,f,q	s	s	s				
1965 Aug 26	s,f,q	s,f,q	s,f,q	s	s	s				
1967 Feb 9	s,f,q	s,f,q	s,f,q	s	s	s				
1969 Aug 26	s,f,q	s,f,q	s,f,q	s	s	s				
1970 Jul 19	s,f,q	s,f,q	s,f,q	s	s	s				
1971 Aug 4	s,f,q	s,f,q	s,f,q	s	s	s				
1971 Oct 3–8	s	s	s	s	s	s				
1971 Dec 7	s	s	s	s	s	s				
1972 Jan 4	s,f,q	s,f,q	s,f,q	s	s	s				
1974 Aug 17	s,f,q	s,f,q	s,f,q	s	s	s				
1977 Aug 29	s,f,q	s,f,q	s,f,q	s	s	s				
1978 Aug 17	s,f,q	s,f,q	s,f,q	s	s	s				
1980 Jul 27–31	s,f,q	s,f,q	s,f,q	s	s	s				
1981 Aug 25	s,f,q	s,f,q	s,f,q	s	s	s				
1983 Sep 8	s,f,q	s,f,q	s,f,q	s	s	s				
1985 Dec 14	s,f,q	s,f,q	s,f,q	s	s	s				
1986 Feb 24	s,f,q	s,f,q	s,f,q	s	s	s				
1988 Aug 29	s,f,q	s,f,q	s,f,q	s	s	s				
1989 Feb 6–10	s,f,q	s,f,q	s,f,q	s	s	s				
1990 Sep 2–7	s,f,q	s,f,q	s,f,q	s	s	s				
1991 Sep 9	s,f,q	s,f,q	s,f,q	s	s	s				
1992 Sep 24–29	s,f,q	s,f,q	s,f,q	s	s	s				
1993 Sep 13	s,f,q	s,f,q	s,f,q	s	s	s				
1994 Nov 2–6	s,f,q	s,f,q	s,f,q	s	s	s				
1995 Oct 5–10	s,f,q	s,f,q	s,f,q	s	s	s				
1996 Oct 23–27	s,f,q	s,f,q	s,f,q	s	s	s				
1997 Sep 17	s,f,q	s,f,q	s,f,q	s	s	s				
1998 Oct 3	s,f,q	s,f,q	s,f,q	s	s	s				
1999 Sep 8	s,f,q	s,f,q	s,f,q	s	s	s				
2000 Oct 10	s,f,q	s,f,q	s,f,q	s	s	s				
2003 Sep 8	d,f,q	d,f,q	d,f,q	d,f	d,f	d,f				
2004 Sep 12	d,f,q	d,f,q	d,f,q	d,f	d,f	d,f				
2005 Sep 16	d,f,q	d,f,q	d,f,q	d,f	d,f	d,f				
2008 Aug 28	d,f,q	d,f,q	d,f,q	d,f	d,f	d,f				
2010 Sep 7	d,f,q	d,f,q	d,f,q	d,f	d,f	d,f	d	d	d	d
2012 Sep 16	d,f,q	d,f,q	d,f,q	d	d	d	d	d	d	d
2014 Sep 9	d	d	d	d	d	d	d	d	d	d
2016 Sep 16	d	d	d	d	d	d	d	d	d	d

Quadrat names (top row) are as per Fig. 2. s – scanned slide, d – digital image, f – shapefile, q – data have been through full QA/QC, and are available in the final processed data set.

**Table 3:** Data availability for the inner flats^12^.

	IF1	IF3	IF2	IF4
1962 Dec 10	s,f	s,f	s	s
1963 Mar 28	s	s	s	s
1963 Jul 5	s	s	s	s
1963 Oct 30	s,f	s,f	s	s
1965 Aug 26	s,f	s,f	s	s
1967 Feb 9		s,f		s
1969 Aug 27	s,f	s,f	s	s
1970 Jul 16	s,f	s,f	s	s
1971 Oct 7	s,f	s,f	s	s
1971 Dec 2	s	s	s	s
1972 Jan 4	s,f	s,f	s	s
1972 Apr 14	s	s		s
1972 Jul 12	s,f	s,f	s	s
1972 Sep 6	s	s	s	s
1974 Aug 17	s,f	s,f	s	s
1976 Sep 27	s,f		s	s
1978 Jan 7	s	s		
1978 Aug 16	s,f	s,f	s	s
1980 Jul 29	s,f	s,f	s	s
1981 Jan 11	s	s	s	s
1981 Aug 28	s,f	s,f	s	s
1983 Sep 9	s,f	s,f	s	s
1984 Nov 22	s,f	s,f	s	s
1985 Dec 9	s,f	s,f	s	s
1986 Feb 24	s,f	s,f	s	s
1988 Aug 24	s,f	s,f	s	s
1989 Feb 7	s,f	s,f	s	s
1990 Sep	s,f	s,f	s	s
1991 Sep 9	s,f	s,f	s	s
1992 Sep	s,f	s,f	s	s
1994 Nov	s,f	s,f		
1995 Oct 4	s,f	s,f		
1996 Oct	s,f	s,f		
1997 Sep 17	s,f	s,f	s	s
2000 Oct 10	s,f	s,f	s	s
2001 Oct 19	s,f	s,f	s	s
2004 Sept 12	d,f	d,f	d,f	d,f
2005 Sept 16	d,f	d,f	d,f	d,f
2008 Aug 31	d,f	d,f	d,f	d,f
2010 Sep 9	d,f	d,f	d,f	d,f
2012 Sep 18	d,f	d,f	d,f	d,f

Quadrat names (top row) are as per Fig. 2. s – scanned slide, d – digital image, f – shapefile. Data have not been through full QA/QC, and are therefore not available in the final processed data set, but can be extracted from the shapefiles once they have been checked for completeness.

**Table 4:** Data availability for the exposed (north) crest^12^.

	NCNE	NCNW	NCSE	NCSW	NCNNE	NCNNW	NRNE	NRNW	NRSE	NRSW	NRNNE	NRNNW
1963 Jan 3	s	s	s	s								
1963 Mar 24	s	s	s	s				s				
1963 Jul 3	s,f,q	s,f,q	s,f,q	s,f,q				s,f,q				
1963 Oct 2	s	s	s	s				s				
1965 Aug 26	s,f,q	s,f,q	s,f,q	s,f,q				s,f,q				
1967 Feb 9	s,f,q	s,f,q	s,f,q	s,f,q				s,f,q				
1969 Aug 26	s,f,q	s,f,q	s,f,q	s,f,q				s,f,q				
1970 Jul 19	s,f,q	s,f,q	s,f,q	s,f,q				s,f,q				
1971 Aug 4	s	s	s	s				s				
1971 Oct 3–8	s,f,q	s,f,q	s,f,q	s,f,q				s,f,q				
1971 Dec 7	s	s	s	s				s				
1974 Aug 17	s,f,q	s,f,q	s,f,q	s,f,q				s,f,q				
1980 Jul 27–31	s,f,q	s,f,q	s,f,q	s,f,q				s,f,q				
1981 Aug 25	s,f,q	s,f,q		s,f,q				s,f,q				
1983 Aug 11	s,f,q	s,f,q	s,f,q	s,f,q				s,f,q				
1983 Sep 8	s	s	s	s								
1985 Dec 14	s,f,q	s,f,q	s,f,q	s,f,q				s,f,q				
1988 Aug 29	s,f,q	s,f,q	s,f,q	s,f,q				s,f,q				
1989 Feb 6–10	s,f,q	s,f,q	s,f,q	s,f,q				s,f,q				
1990 Sep 2–7	s,f,q	s,f,q	s,f,q	s,f,q				s,f,q				
1991 Sep 9	s,f,q	s,f,q	s,f,q	s,f,q				s,f,q				
1992 Sep 24–29	s,f,q	s,f,q	s,f,q	s,f,q				s,f,q				
1993 Sep 13	s,f,q	s,f,q	s,f,q	s,f,q				s,f,q				
1994 Nov 2–6	s,f,q	s,f,q	s,f,q	s,f,q				s,f,q				
1995 Oct 5–10	s,f,q	s,f,q	s,f,q	s,f,q				s,f,q				
1996 Oct 23–27	s,f,q	s,f,q	s,f,q	s,f,q				s,f,q				
1997 Sep 17	s,f,q	s,f,q	s,f,q	s,f,q				s,f,q				
1998 Oct 3	s,f,q	s,f,q	s,f,q	s,f,q				s,f,q				
1999 Sep 8	s,f,q	s,f,q	s,f,q	s,f,q				s,f,q				
2000 Oct 10	s,f,q	s,f,q	s,f,q	s,f,q				s,f,q				
2003 Sep 8	d,f,q	d,f,q	d,f,q	d,f,q				d,f,q				
2004 Sep 12	d,f,q	d,f,q	d,f,q	d,f,q				d,f,q				
2005 Sep 16	d,f,q	d,f,q	d,f,q	d,f,q			d,f	d,f,q	d,f	d,f		
2008 Aug 28	d,f,q	d,f,q	d,f,q	d,f,q	d,f	d,f	d,f	d,f,q	d,f	d,f	d,f	d,f
2010 Sep 7	d,f,q	d,f,q	d,f,q	d,f,q	d,f	d,f	d,f	d,f,q	d,f	d,f	d,f	d,f
2012 Sep 16	d,f,q	d,f,q	d,f,q	d,f,q	d,f	d,f	d,f	d,f,q	d,f	d,f	d,f	d,f
2014 Sep 9	d	d	d	d	d	d	d	d	d	d	d	d
2016 Sep 16	d	d	d	d	d	d	d	d	d	d	d	d

Quadrat names (top row) are as per Fig. 2. s – scanned slide, d – digital image, f – shapefile, q – data have been through full QA/QC, and are available in the final processed data set.

**Table 5:** Data availability for the exposed pools^12^.

	P1	P2
1962 Oct 13	s,f	s,f
1963 Mar 24	s	s
1963 Jul 3	s	s
1963 Oct 2	s,f	s,f
1965 Aug 26	s,f	s,f
1969 Aug 26	s,f	s,f
1970 Jul 19	s,f	s,f
1971 Aug 19		s
1971 Oct 6	s,f	s,f
1972 Jan 4	s	s
1972 Jul	s,f	s,f
1974 Aug 17	s,f	s,f
1978 Sep 1	s,f	s,f
1980 Jul	s,f	s,f
1981 Aug 28	s	s,f
1983 Aug 11	s	s
1983 Sep 8	s,f	s,f
1984 Nov 23	s,f	
1985 Dec 12	s,f	s,f
1986 Feb 29	s	s,f
1988 Aug 29	s,f	s,f
1990 Sep	s,f	s,f
1991 Sep 9	s,f	s,f
1992 Sep	s,f	s,f
1994 Nov	s	s,f
1995 Oct 6	s,f	s,f
1997 Sep 17	s,f	s,f
1998 Oct 3	s,f	s,f
1999 Sep 8		s
2000 Oct 10		s
2004 Sept 12	d	d
2005 Sept 16	d	d

Quadrat names (top row) are as per Fig. 2. s – scanned slide, d – digital image, f – shapefile. Data have not been through full QA/QC, and are therefore not available in the final processed data set, but can be extracted from the shapefiles once they have been checked for completeness.

**Table 6:** Description of data present in the final processed data set.

Column	Description
Quadrat	Quadrat name as per Fig. 2.
Year	Year of survey
Colony ID	Unique identifier for each colony, maintained through time
Species	Species code^12^.
Edgestatus	0 if entire colony lay within quadrat, 1 if part of the colony was outside the quadrat (and not digitised)
Area	Area of the colony in square cm.

## Technical Validation

The first step of the data validation occurred in the field, with the taxonomic expert present double-checking each mud map to ensure that all colonies had been included and were consistently identified. Data for the protected and exposed crests have also been through a number of computer-based quality control steps described below to ensure that they are reliable. This quality control process has not been completed for the inner flat or the exposed pools, and hence only images and shapefiles are presented for these two sites. It is strongly recommended that before any data is extracted from these files, the steps described below are followed.

All digitising and taxonomic identifications were checked, and where necessary, refined by the lead author to remove inconsistencies between the multiple operators who undertook the initial image processing. The initial step involved closely checking each quadrat to ensure colony outlines were correctly located, and that all colonies had been included both according to what was visible in the image, and what was recorded in the field on the mud map. Sequential pairs of digitised images were then compared side-by-side to ensure any colonies recorded as lost were not present in the later image, that all new colonies were not present in the earlier image, and that species identifications were consistent. A second taxonomic check involved creating a pivot table of colony number against year, using the sum of species number. The average of the sum of species number across years was then subtracted from the species number. Any difference from zero indicated either changes in the species assigned to the colony over time, or multiple colonies assigned the same number. The digitised images were then carefully checked to resolve discrepancies. In the process, all coral taxonomy was updated to that presented by Veron^13^. There has been no revision to incorporate recent taxonomic changes. This validation has only been undertaken for corals and other benthic invertebrates. No validation has been done for algae, and thus data for these should not be relied upon without further work.

As species level taxonomy could be difficult to distinguish in the images for some species, to ensure that changes in community composition over time were not related to changes in taxonomy, the data set has been analysed by pooling species into groups that could potentially be mistaken for each other. This produced identical ordination plots to those produced for the species level taxonomy at both the exposed and protected crests^1^, providing evidence that there are no major taxonomic inconsistencies across the data set for these two sets of quadrats.

## Data Availability

No computer code was used to generate this data set.
